# Retrospective View of the Late Improvements in Medicine and Surgery

**Published:** 1817-07

**Authors:** 


THE LONDON

### Medical and Physical Journal.

1 OF VOL. XXXVIII.] JULY, 1817. [NO. 221.

"For many fortunate discoveries in medicine, and for the detection of nume-
"rous errors, the world is indebted to the rapid circulation of Monthly
"Journals; and there never existed any work to which the Faculty in
"EUROPE and AMERICA were under deeper obligations than to the
"Medical and Physical Journal of London, now forming a long, but an
"invaluable, series."—RUSH.

### *Retrospective View of the late Improvements in Medicine and Surgery.*

Quid ego et populus mecum desiderit audi.

WE have received an intimation that some of our readers expect a RETROSPECT. After the pains we have taken to keep up with every improvement in the various branches of medicine, we must suppose that this hint is from some of our younger readers, who are desirous of an analysis made ready to their hands. We shall, therefore, take this opportunity of again addressing our friend JUVENIS, and if, in the course of our remarks, we should offer any thing to amuse the more experienced practitioner, we may truly say,

"Indocti discant, ament meminisse periti."

We are ready to confess there are two subjects with which we have been unwilling to fill our pages. These are the more complicated researches of philosophic chemistry and the reveries of physiologists. The first, if at all, is only distantly connected with medicine. From the second, the practical physician would rise with disgust, and the learner, either filled with vanity, or alarmed at the necessity of learning what he finds utterly beyond his comprehension.

Chemistry, as far as concerns therapeutics, has never escaped our notice, and, on the more recondite parts of that bewitching science, we have been careful to present the result of the labours of others without vouching for the accuracy of their experiments or the fairness of their inductions. Animal chemistry, though little connected with the common operations of animal life, has never been overlooked by us. In a retrospect, we know not how to add more, excepting by showing the errors into which its devotees are led.

"Chemists (says an ingenious writer,) have discovered long ago, that the elements composing organized substances are comparatively few in number, and such as are well known to exist in great abundance in the inorganized state. The four chief of these are oxygen, hydrogen, carbon, and azote, to which may be added lime, magnesia, phosphorus, sulphur, and iron; but the two last of these exist only in very small proportions. It is not so easy to demonstrate, that the usual inorganic agents* are employed and directed by the vital principle in its operations, though the following arguments will perhaps render it probable that this is actually the case in many instances, if not in all. 1. These agents are universally diffused through nature, and are so intimately connected with matter in every form, as to become, as it were, a part of itself, and to be inseparable from it by any means within our power or conception. In short, they make inorganized substances what they are, and give origin to, and constitute the very essence of the laws which govern them. Further, we know that caloric, and perhaps light, are absolutely necessary to the existence of all organized beings. We cannot, indeed, say the same so easily of the electric fluid, but we know that some animals, for example, have the power of accumulating this fluid in organs destined by nature for the purpose, and that all animals are very readily and strongly affected by it; from which we may infer at least, that it exists invested with all its powers in *some* organized beings, and that there is a possibility of its existing in *all*."

We shall not detain the reader longer than to remind him of hybernating animals, which exist so long without light, and even without heat, but of their own creation; and that some of these, being what are called cold-blooded animals, have so little power of creating heat as to take their temperature from the atmosphere or surrounding bodies. It is true some animals have a power of exciting electricity.

"* By these I mean caloric, light, the electric fluid, that power or principle (if it be different from the electric fluid) which is the cause of common chemical action, &c."

*Some*

*Some* animals have also a power of secreting a poison for their defense, others a turbid fluid: *ergo*, "there is a possibility of its existing in *all*."

We admit, however, that this hint concerning electricity or galvanism, as a means of secretion is not peculiar to this author, nor even to the adepts in chemistry.

"Again, (continues the same writer,) we see that the usual agents of inorganic matter *do* very frequently operate in organized beings: thus, in the instances of phosphate of lime, &c. before mentioned, they evidently operate, because these substances cannot be produced by any other means, except we suppose that the vital principle exerts a similar and equivalent power, which is superfluous and contrary to all the principles of reasoning. Since, therefore, these agents and the laws they prescribe to inorganic matter are so intimately connected with it, and are found to be actually employed in many instances as subordinate agents by the vital principle, (facts which sufficiently prove their aptness and adequacy for that purpose,) we may easily conceive them to be employed in every instance, more especially *as we cannot form an idea of any agent that would be better qualified for the purpose*. 2. If the usual agents of inorganized matter have no share in organic operations, we must either suppose that the vital principle itself operates immediately upon the material inorganic elements, or that it operates through the medium of other subordinate agents than those which naturally govern them. The first supposition will give to the vital principle a power little short of a creative one. It is similar, for example, to supposing that a potter can act at once upon a crude mass of clay, and convert it into a beautiful piece of highly finished porcelain, without the aid of tools, or the intermediate processes of tempering, baking, &c. Besides, if the vital principle were the only agent that operated in organic beings, the moment it ceased to act, the *vinculum* that kept the whole together would necessarily be removed, and an immediate dissolution would be the consequence, which is not the case: for, though it be true that all organic substances sooner or later decay, yet this does not take place with that rapidity which we should expect from an object so differently constituted as one formed by the sole agency of the living principle upon inorganic matter must necessarily be. As to the second supposition, it is involved in the same difficulties as the first, for as the subordinate agents would necessarily be dependent for their existence upon the vital principle, it is evident, that when the vital principle ceased to exist, they must cease to exist also, and an immediate dissolution of the organized being would take place as before.

"Having thus attempted to show, that the immediate agents and elements which act and are acted upon in organized beings, are nothing more or less than the agents and elements of inorganic nature, directed and modified by the vital principle, we come now (says this writer,) to consider more particularly the manner in which the vital principle operates in the production of organized beings."

Now, in compassion to our younger readers, whose brains may be addled by a complexity of sentences and phrases which it must be admitted are simple enough taken by themselves, we shall concede all that is said of the potter and his clay, of the vital principle, the creative power, the agents, whether organic or inorganic, and even admit that WE *cannot form an idea of any better qualified agents*;" last of all, "that these machines all terminate in parts infinitely minute, and in these minute terminations only that all changes immediately take place, by which means their nature is totally excluded from our observation." [That is, that we know nothing about the matter.—Would the author, for his own sake, had stopped here.] "We observe, however, (continues he,) that one grand *principle* pervades them all, which is *comminution*." This we concede also, and that "one and one make two, whether the author of the addition be an idiot or a philosopher, or even the Deity himself." But we confess ourselves much at a loss to know what are the inferences to be drawn from such premises. Probably such might be the case with the writer, for instead of inferences, he contents himself with a few general expressions concerning the ability of the vital principle.

"The vital principle, (we are told,) is enabled to operate through the medium of the nerves upon each individual particle of the blood, as it passes under its cognizance, and to determine upon those elements which require to be separated, and subsequently removed by the absorbent system; while the remaining elements are left to unite *suâ sponte*, and in virtue of their own proper and natural affinities, and thus to form the new product required.* Hence, organized substances

"* We may suppose with some physiologists, that in the formation of organic products, the vital principle employs the agency of the substances in general might no doubt be formed artificially, if we could bring together the elements composing them precisely in the same manner in which they are brought together by the organic process."

Let not our young reader be alarmed at all this, nor suppose that he is unfit to practise physic 'till he understands how those animals are supported who have no nervous system to "take cognizance of each individual particle of blood;" For these he may conclude that the "*sua sponte*" affinities will be sufficient. Let him, however, be very cautious how he attempts to "bring together elements in the manner they are brought together by the organic process, so as to form organized substances!" For he may be assured, that before he, or this chemico-arithmetical physiologist were born, one and one made a third. What follows will explain this matter better; and the commencement of the succeeding paragraph gives us a fair opportunity of concluding the chemico-physiological part of our Retrospect.

"Thus then, (continues the author,) we consider the vital principle as the immediate *cause,* not the *effect,* of the organic process: as a power or principle endued by the Creator, with a faculty little short of intelligence, by means of which it is enabled to construct such a mechanism from material elements and agents, as to render itself capable of taking further advantage of their properties, and of making them subservient to its use. But it will be doubtless objected, that it is equally difficult to conceive how the vital principle can construct this machinery, as it is to conceive how it can use it when constructed. This must be admitted. It is often as difficult for the chemist to prepare his apparatus, as it is to make his experiments; but the experiments cannot be made without the apparatus. In like manner, one part of an organized being is constructed by

---

the electric fluid, by means of which we can conceive it to be able, by placing the elementary particles in different states of electricity to separate some, and thus favor the union of others, according to the ends it may have in view. It must, however, be observed, that many organic substances are liable to great and extraordinary changes in external characters at least, without any apparent increase or diminution of their material elements, as, for example, fibrin and albumen."

the the agency of another, these again by others, and so on; for no operation can take place without its appropriate machinery. But the original power which appears at present to contrive and regulate these operations, was, as before observed, created by the Deity, and not only gifted with these faculties, but with the additional one also of having its existence in union with material elements and agents, perpetuated by communication from one individual to another, which is the only manner in which organic beings can be produced.

"In these preliminary remarks, the reader, perhaps, will find little new."—In this last remark we perfectly agree with the author.

Thus much in excuse for our inattention to chemistry as connected with physiology. But some greater apology may seem necessary for our apparent inattention to physiological theories founded on no other facts than such as may be traced in a living animal.

Darwin and Brown were both ingenious men, and arrived, as we shall presently see, at the same point by different routs. Another author, we are informed, met Bichât in his road; and we suspect that in this road all other men will meet in perfect harmony. The author refers us to the 520th and following pages of his Treatise on Fever, published anno 1799, for some of his opinions.

"That excitement (says he) which is followed by exhaustion is always healthy. The debility it occasions affects no parts of the system but those on which the animal functions depend, and in them only occasions debility by diminishing their excitability, in consequence of which they cease for some time to be excited, and the animal functions are suspended, that is, sleep takes place; during which, the excitability of the organs concerned in these functions gradually increasing, they become sufficiently sensible to be again roused to action by the usual stimuli. The same stimuli again in a short time impair their excitability, which is again in like manner restored; hence the constant alternation of vigilance and sleep. During neither of these states are the powers of life at all impaired. The system is in as perfect health in a state of exhaustion as in that of moderate excitement. The powers by which the body is preserved do not partake of this alternation; it is confined to those powers, by which *the animal is connected with the external world,* by which he perceives and acts.

"The latter set of powers cannot exist independently of the former, but the former may without the latter. We have reason to believe that this is the case in the less perfect animals, which seem to possess no powers but those on which their existence depends; and which may be regarded as the link that connects the more perfect animal with the vegetable world.

"We have ample proof, that, even in the most perfect animals, the vital and natural functions may be in a state of perfect vigour, where the powers on which the animal functions depend never existed. Human and other fœtuses have been born without the head, in other respects well-formed and of the full size. Such a fœtus dies as soon as a ligature is thrown around the umbilical chord, because the blood no longer undergoes that change which is caused by the vicinity of the maternal blood in the placenta, and which is effected, after birth, by respiration; a function, which, depending on the nervous system, cannot be performed by an animal without the brain. Could such a fœtus after birth be made to respire and be supplied with nourishment, we have every reason to believe, from what we know of the animal œconomy, that it would live and increase in size after birth, as it does before it; but it would certainly experience no alternation corresponding to that of vigilance and sleep in the more perfect animal."

Now, if we understand all this, the first part is only the road in which Brown and Darwin met, the one with excitability, the other with sensorial power;* and each teaches us, that,

---

* "Now, as the sensorial power, or spirit of animation, is perpetually exhausted by the expenditure of it in fibrous contractions, and is perpetually renewed by the secretion or production of it in the brain and spinal marrow, the quantity of animal strength must be in a perpetual state of fluctuation on this account; and if to this be added the unceasing variation of all the four kinds of stimulus above described, which produce the exertions of the sensorial powers, the ceaseless vicissitude of animal strength becomes easily comprehended.

"If the quantity of sensorial power remains the same, and the quantity of stimulus be lessened, a weakness of the fibrous contractions ensues, which may be denominated *debility from defect of stimulus.*

that, when an animal is wearied, he is refreshed by sleep, and may go to work again—that, when he has laboured long in body or mind, he must rest—and, lastly, that he must neither sleep too much nor work too hard. It is true, the author just quoted is to us a little obscure, when he thinks it "necessary to premise, that, by the term exhaustion, he means the state in which the body exists during sleep"—we find *our* bodies recruited during sleep!

We admit, indeed, that Dr. Wilson and Bichât differ in many points, but suspect that most readers will discover a similarity of language when the former speaks of "powers by which an animal is connected with the external world."

---

*stimulus*. If the quantity of stimulus remains the same, and the quantity of sensorial power be lessened, another kind of weakness ensues, which may be termed *debility from defect of sensorial power;* the former of these is called by Dr. Brown, in his Elements of Medicine, direct debility, and the latter indirect debility. The coincidence of some parts of this work with corresponding deductions in the Brunonian Elementa Medicinæ, a work (with some exceptions) of great genius, must be considered as confirmations of the truth of the theory, as they were probably arrived at by different trains of reasoning."—See *Darwin's Zoonomia*, vol. i. p. 96, 3d edit. 1801.

"Life then, or those functions which we call living, are the effects of certain exciting powers acting on the excitability, or property distinguishing living from dead matter. When these effects, viz. the functions, flow easily, pleasantly and completely, from the action of these powers, they indicate that state which we call health.

"We may, therefore, as we before hinted, distinguish three states of the irritable fibre, or three different degrees of excitability, of which the living body is susceptible.

"1. The state of health which is peculiar to each individual, and which has been called by Haller, and other physiologists, the tone of the fibre. This is produced by a middle degree of stimulus acting upon a middle degree of excitability: and the effect produced by this action, we call excitement.

"2. The state of accumulation, produced by the absence or diminished action of the accustomed stimuli.

"3. The state of exhaustion, produced by the too powerful action of stimuli."—*Garden's Lectures on Zoonomia, p.* 197.

As we have already given our opinion very much at large on the French philosopher,* our readers will not expect us on the present occasion—*Tantos componere lites!* Few people will question whether secretion and every other property of an animal not controlled by the will, nor always the object of his senses, exist, whether he sleeps or is awake. But there is a difficulty in the language of the English philosopher which we confess ourselves unable to solve. Which are the imperfect animals that exist "without those powers which connect them with the external world?" How do they procure their food? Probably the author may refer to hydatids and other parasite animals, but this should be explained.

Another objection we make, is to certain inferences on subjects which admit of proof. To ascertain how an animal of the class accustomed to have a head can exist without one, it is not necessary to guess whether, under certain circumstances, an acephalous fœtus could live and encrease after birth, nor would it be easy to make an experiment on a monster whose deviations in other respects might be uncertain. Instead of this, we would recommend that an animal should be decapitated, or pithed, that the lungs should be alternately inflated and emptied, and food injected by the œsophagus. Query—How long will such an animal live and grow? Not long, it may be urged; because no heat is excited, and the secretions cease. But may not all this be remedied by Galvanism?

We cannot dismiss this subject without that attention to language, in which we think it our duty never to remit. The writer we have in view seems to consider Bichât's distinction between animal and organic life as synonymous with the nomenclature of animal, vital, and natural functions. To us, the first of Bichât's terms seems applicable to animals only, the second to vegetables in common with animals. But we are at a loss to know how to explain the other three. All functions must be vital, as they only exist during life; and all must be natural, as they exist only by the laws of nature. These three terms, if their order is inverted, appear

* See Lond. Med. and Phys. Journal, vol. xxv. p. 41 and 125.

to us very similar to those adopted by Linnæus to comprehend the whole Systema naturæ. *Lapides crescunt, vegetabilia crescunt et vivunt, animalia crescunt, vivunt et septiunt.*

We shall, perhaps, be accused of having dwelt too long on topics so unimportant. For this we have already made our apology, and may now add, that such subjects are perpetually obtruded on the public, and the attention to them, we are convinced, proves a considerable retardation of progressive improvement in a most important science. For this reason, we have not hitherto attempted to defend ourselves from some animadversions in a most respectable journal, which we could not doubt were directed to us:—we are the more ready to do it now, as it may prove the means of introducing a subject the importance of which cannot be questioned, and to which we have devoted several pages in this and in our preceding Number. We can assure our candid and very respectable brethren, that, when we acknowledged our incapacity to understand Dr. Carson, we spoke only our real feelings; and, we regret to say, such is our dulness, that, notwithstanding all that has been written by way of elucidation, we still remain in the dark. We are glad to find, however, that, in the "cause of the blood's motion through the arteries, Dr. C. does not materially differ from other writers." If we understand the question, his discovery is in "the manner in which the blood enters the heart from the vena cava." To account for this, some hydrostatic laws are proposed, explaining the effect of the *resilience* of the lungs, the vacuum in the heart, and the law by which all fluids find their level. We are by no means certain that we are right in these positions; because it is well known that the blood circulates in animals that have no lungs and no heart,* consequently no *resilience* and no vacuum. Nay, we are assured that, in animals whose lungs are formed like the human, the circulation will continue ten or fifteen minutes after respiration has ceased. We are aware that some of the advocates for *resilience* claim it only

* See Hunter on the Blood, p. 133, chapter on the Heart.

as as an auxiliary power:* hence it may be urged, that the heart can do without it for a quarter of an hour or so. Without inquiring into so complicated a subject, we shall just copy, from the same writer, Dr. Carson's manner of explaining that process, which is now pretty generally called "the adhesive inflammation."

"When a wound is made in the body, and the parts brought together and kept in that state, the plastic nature of the blood that had oozed out of the arteries drying near the surface, unites the edges of the wound; but the blood oozing out of the arteries remains liquid. The veins in the mean time have become emptied, in consequence of the blood which they contained having taken the less resisted course, and returned to the heart. The blood, then, which had flowed from the mouths of the arteries into the interstices between the divided surfaces, being less resisted towards the mouths of the veins than in any other direction, necessarily enters the veins and continues its course to the heart. Other blood follows, and thus a communication is established between the artery and the opposite vein. The blood forming this slow current coagulates upon the external surface, and in due time a vessel is thus formed between an artery on one side and a vein on the other of the wound."

Now, is this intended as a riddle, or is it a description of the properties of the blood to coagulate, and, if it retains its life, to unite parts which were previously divided?—all which has been so minutely described by the most accurate physiologist of the last century.

We are now led to consider the experiments of a physiologist who has done much to improve the healing art, and whose pathological doctrines we have always treated with respect. By this gentleman's experiments, it appears that an artery has no pulsation, excepting when external pressure is applied to it. We are not disposed to do more than express our doubts whether the experiments hitherto made are

---

* A kind of "suction influence," another journal calls it, "which explains, and which only can explain, why a punctured vein does not bleed unless a ligature is thrown upon it, between the puncture and the heart." If this were established with certainty, how much trouble might be spared in bandaging an arm after bleeding! See *Annals of Med.* p. 167.

conclusive. We have been told that, in the artery of a dog, mere exposure is enough so far to alter its action as to induce a contraction almost equal to obliteration. This experiment has been confirmed by another writer. "It is singular," says he, "that, on exposing arteries, a contraction of a small portion, sometimes forming a mere ring, frequently took place, with a corresponding augmentation of the part just above." Now, this is exactly what we should expect if an artery, by exposure, is deprived of its *vasa arteriarum*, which, it is well known, are derived from the surrounding parts. In this condition, as we observed in our last Number (see pages 502 and 503), there is an imperfection in the artery, which (to use a language we find most writers are at last obliged to adopt) may induce the artery to act as if "from a consciousness of its own condition." Thus, without being aware of it, we fall into another mode of expressing Mr. Hunter's "stimulus of necessity;" that is, the new condition of the parts, and the danger to which the animal is exposed, proves a stimulus to a new mode of action, and, under this new mode of action, the pulsation may cease.

We have offered this hint to show how many minute circumstances are often overlooked, even by accurate philosophers, in making experiments on living animals. A still more important consideration, however, is within our view. By the experiments which have been lately made on arteries, we have reason to believe that their contractile powers is much greater than is generally supposed. We are aware that this is not so well ascertained in the human subject, yet Mr. Warner succeeded in stopping the bleeding of divided arteries in the leg and fore-arm without ligatures.*

Thus

* We recollect a tradition, that, in those days, government proposed experiments to be made on the larger arteries of condemned criminals. The surgeons very fairly felt themselves lessened by being made executioners, and demanded the liberty of taking up the arteries, if the agaric should prove unequal to stopping the flow of blood. This was refused, and the criminals were liberated. In these more enlightened days, might not some criminals who have forfeited

Thus have we somehow brought ourselves to a real Retrospect; and, in so doing, we trust, have convinced our readers how completely retrospective we have always been. This subject will again occur in our critical department, in order that our readers not only may be enabled with greater facility to comprehend the whole, but led to compare and reason for themselves. We shall anticipate some of Mr. Bell's remarks on the ligatures of arteries, reserving our general analysis of his work till the whole volume is completed.

After several observations on some "ingenious trifles which have been offered as improvements," and "on what he was taught both in the dissecting room and the hospital, his early experience in preparing for his injections," &c. Mr. B. proceeds to a detail of "what is essential to the ligature on an artery." This is afterwards summed up in the following paragraph: "There are thus four changes, 1. The obstruction of the artery by the ligature. 2. The formation of a clot behind the ligature. 3. The discharge of coagulable lymph by the inflamed coats. 4. The final change by which these coats degenerate into a common texture." All these are exhibited in the plate, and the examples are taken from the human subject."—Now, if we understand Dr. Jones, great care must be taken that the artery is not permanently obstructed, if justice is done to his operation; and, if this is really the case, the clot of blood and the discharge of coagulable lymph appear to us both equally unnecessary, or are provided for without the necessity of obstructing the artery by ligature. Indeed, Mr. Bell gives an instance in which the carotid of a dog was obstructed by a cord put loosely round: an engraving shows the same vessel in an ass obstructed in a similar manner. "The ligature lies quite loose, was never drawn, consequently never cut the coats of the artery; an incised wound could, therefore, not have been

---

forfeited their lives by the severity of our laws be selected, to see how far an artery will contract by mere exposure, or what other means might be used to lessen the most painful and most dangerous part of many operations.

made made here, nor could the blood be mechanically obstructed by the ligature, yet the artery is obstructed, the effect of the ligature is perfect." If such is the case, and we are not disposed to doubt it, where is the necessity for "obstructing the artery by a ligature;" or what proof have we that the coats are inflamed in order to throw out coagulable lymph?

As Mr. Bell's performance will come before us again, we should dismiss it at present were it not for a remark or two on his language very much to our purpose. Where did he meet with the expression, "an artery's stimulus to its perfection," or what are his motives for adopting, with apparent reluctance, "what is called in the present language of surgery the adhesive inflammation?" If these expressions are used as the most appropriate, it would have been handsome to have given the inventor credit for them. But, in our opinion, one of them is improperly applied, and the other hastily assumed: 1st. As far as has hitherto been discovered, the artery contracts, as the blood coagulates, from the stimulus of necessity. The artery was previously as perfect* as the nature of its functions required, but now it is unequal to those functions, and, in order to preserve the animal, contracts till it is gradually obliterated. 2ndly. There have hitherto been no proofs that the adhesive inflammation is at all necessary for the union of the sides of the artery; though Dr. Jones discovered an effusion of lymph when he cut through the inner coats of an artery; yet that process could not be the cause of its obliteration, as blood afterwards circulated through it, and it has even been found that contractions have taken place where no coagulated lymph was discovered.

Thus have we been insensibly led into the retrospect of surgery before medicine. We have but few other remarks

---

* The *stimulus of perfection* is a term used by Mr. Hunter to signify those actions by which an animal arrives at the highest degree of perfection of which his nature is capable. Of this kind are the descent of the testicle, and the various changes which occur at puberty, &c. An action is induced by the *stimulus of necessity*, when the animal's life is endangered, and only saved by such an action.

to to make on the former, the principal improvements being on the means of preventing hæmorrhage, either from accident, disease, or operations. We have taken much pains to give this subject a new turn, and to direct the inquiries of the philosophic practitioner to the powers of arteries, and of the blood, and to the stimuli under which they assume new actions. Instead of disputing about the vitality of the blood, or the apparent consciousness of vessels, we have only requested that the changes in the form and action of each may be duly attended to. Whenever this has been done, however imperfectly, we constantly find every candid inquirer adopting a similar language, which, if not admitting, at least implies life and consciousness. Let us ask too, is there any thing more extraordinary in all this than in the new character assumed about this season of the year by whole races among the inferior animals? Are not both sexes irresistibly directed to the propagation of the species? In the finny and feathered tribes, is not the formation of certain parts changed preparatory to such a process? Does not the female bird learn to forego the use of her wings, and almost to exist without food? Do not both sexes learn to construct a nest, and are they not endowed with a courage which seems at variance with their character at every other period of their existence?—When we constantly witness such provision, why should we feel surprised if a fresh wound is united without the agglutinant traumatics, of which now only the names remain; or, if that process fails, that it should suppurate without digestives, granulate without incarnatives, and cicatrize without eccoprotics?—And, if such is the case, what is there more extraordinary in the contraction of vessels, or the coagulation of the blood, when those processes are necessary for the immediate preservation of the animal.

Surgery has, however, been further improved by the treatment of diseases in their most complicated forms. The milder mode of uniting sinuses does much honour to the restorer and improver of this practice, and the boldness with which the knife has been introduced into the complicated articulations of the tarsus and metatarsus, as well as the larger joints, shows a progressive improvement in the art to to which it is impossible to fix any limits. To these we might add the improvements in the diagnostic and consequent treatment of various complaints in the urinary and genital organs, the effect of anatomical investigation, the description of which is assisted by the graver or etching tool.

In midwifery, which must ever be considered a part of surgery, we trust our unembarrassed review of Dr. Stewart, may be a means of arresting the mischief with which, in the opinion of the most experienced practitioners, his book was fraught.—If so, a crude performance of that kind may not be without its utility. It may excite inquiries, or at least show that, however unnecessary or improper large doses of opium with stimulating dietetics may be, they are not always fatal, even when their danger is increased by unnecessary manual assistance.—Our next number will contain a drawing of an important improvement in the instrumental part of midwifery, sanctioned by the ablest practitioners of the age.

The improvements in medicine have been much more considerable, and, in our opinion, much more important than in surgery. We hope we may now congratulate our countrymen, and perhaps the world at large, on having in great measure risen above that unfortunate artificial arrangement of fevers which has so long proved destructive in private life, and of which, in the army and navy, we may truly say,

Πολλας δ'ιφθιμȣς ψυχας αϊδι προιαψεν
Ηρωων——.

Dr. R. Jackson first led the way to this improved practice. His language, it may be objected, was too much polished, and, it must be confessed, some of his theories were such as could not be reduced to proof; yet this ought not to have superseded a due attention to his facts, which were incontrovertible. Happily they have led to a rational and successful practice. We have sometimes expressed our doubts, whether this practice may not be continued when in the British Island, at least a change in the constitution of the atmosphere, may produce a change in the character of disease. There is, however, little danger of this in tropical climates, and if Dr. J. relieved medicine from the influence of a nomenclature which has done so much to impede its progressive improvement, thousands now alive, and of the succeeding generation, ought to bless his memory. Dr. Bancroft's ponderous volume has succeeded much better with the public.

It is not often that much time elapses without something being said systematically on fever. Dr. Armstrong has published an able and well-written volume on this subject, and, although he still retains the former names and definitions, and divisions, of febrile affections, he has had sufficient courage to depart from the beaten track in pursuing his curative indications,—how far we agree or differ from this writer will be seen by turning to our analytical review of his publication. We regret that so much pressing matter has prevented our notice of Dr. Mills, and several other writers, on the same subject.

Though Dr. Bancroft's notions of contagion are more confused than any we have met with, he is still decided in the non-contagious property of yellow fever. The subject of contagion is, indeed, so necessarily connected with fever, that most of the writers on the former have been drawn into a consideration of the latter. Among these we have particularly noticed Dr. Dickinson, and another writer in the Edinburgh Journal. These, it is true, only speak incidentally of contagion, but both are remarkably clear in every important particular, whether diagnostic or practical.

Some writers have lately directed their sole attention to contagion. We have a pledge of long standing, which we are often called upon to redeem, and for which we are collecting materials from every quarter. The American writers have large opportunities of investigating this question in a new country where disease assumes those formidable features which the extremes of climate, suddenly encreased population, an extensive coast, and new land frequently turned up, afford in all their varieties. Yet we are forced to acknowledge, that the science has not hitherto improved in that quarter since the death of the much-lamented Rush.

We have before had occasion to remark the unsatisfactory manner in which Dr. Hossack has treated the subject, and his strange assumption of a certain *tertium quid* from an author in whose writings we have never been able to discover any thing which could lead to the adoption of such terms.

We have already hinted how much the practice in fevers has suffered by a premature nosology. Must we still have to complain, with Mr. Bakewell and Sir Humphrey Davy, that " the avenues to knowledge are barricadoed by a fortification of hard words and frivolous distinctions ?" Before we do this, we must take a closer view of Mr. John Mason Good's Physiological System of Nosology, which is, unquestionably, the most learned and laborious of any of the medical publications that have lately issued from the press. The whole " form and substance" of the book is, indeed, more like the production of a German literatus than of an English general practitioner. The author, after stating his objections to former nosological systems, and pointing out the extent of coincidence between himself and another, who, by his *radius*, undertook to describe all medicine and its writers, proposes to divide the whole of morbid phenomena into the classes Cœliaca, Pneumatica, Hæmatica, Neurotica, Genetica, Eccritica, and Tychica ; each class being divided and subdivided into numerous orders, genera, and species. It cannot be denied, that Mr. Good has evinced a great deal of ingenuity, as well as industry, in endeavouring to give connexion, shape, and beauty, to such materials. Nosology, we have repeatedly said, is labour lost, when it goes upon the presumption of our capacity to define diseases as the botanist delineates the parts and peculiarities of a plant. Mr. Good's plan, however, of naming affections according to the part or organs affected, is less objectionable than the nomenclature of many of his predecessors, insomuch as the announcement of the diseased state involves less of hypothesis with regard to diseased production. The notes and occasional observations of Mr. Good are often exceedingly instructive ; and, although we do not predict that the new arrangement proposed will be pursued by teachers of medicine, we do believe that the volume in question will find its way generally into the libraries of medical amateurs.

We have lately noticed two other writers on nosology. One confines his remarks to cutaneous diseases, which, being the objects of our external senses, seem to offer the fairest subjects for artificial arrangement. Yet, when we consider how much even these diseases are modified by season, constitution, and even the various stages through which they pass, and at the same time reflect on the difficulties attending such arrangement, when confined to original conformations in the various and less complicated operations of nature, we must surely confess, that our progress in the diagnostic of disease is much too imperfect to attempt the substitution of *character* for description.

Willan has the merit of first attempting to assist arrangement by elaborate descriptions and by coloured engravings; but his descriptions are rendered complicated by the unnecessary and indiscriminate production of authorities, and by his attempts to reconcile them, instead of boldly deciding for himself, or acknowledging his incapacity so to do. Dr. Bateman completely gave up the point, when he told us, in his first edition—

" Most of the writers, who have composed express treatises on cutaneous diseases, in modern times, have implicitly adopted the nomenclature of the ancients, without attempting to render it more definite, or to improve upon the diagnosis which they had pointed out. The essays of Mercurialis, Hafenreffer, Bonacursius, and Turner, were written after this manner; and even Lorry, in his able and elegant work, does not step far out of the ancient path. About the year 1780, however, an elaborate classification of the diseases of the skin was published by Prof. Plenck, of the University of Buda; and, subsequently to the commencement of Dr. Willan's publication, a sort of arrangement has been proposed, in the splendid and pompous performance of M. Alibert, which however is altogether destitute of method, except in the distinctions of the species under each genus.

"The arrangement of Plenck is founded upon the same principles as that of Dr. Willan, namely, upon the external appearances of the eruptions: but, in filling up the scheme, he has deviated widely from the strict laws of classification, which naturalists have established. Nine of his fourteen classes very nearly correspond with the eight orders of Dr. Willan. These are, 1. Maculæ; 2. Pustulæ; 3. Vesiculæ; 4. Bullæ; 5. Papulæ; 6. Crustæ; 7. Squamæ; 8. Callositates; and 9. Excrescentiæ. But the five remaining classes comprise, 10. Ulcera; 11. Vulnera; 12. Insecta cutanea; 13. Morbi Unguium; and 14. Morbi Capillorum, which are less judiciously devised. But such a classification must fail to answer its end, because it requires the different stages of the same disease to be considered as so many distinct maladies, and to be arranged in several classes."

Yet, after these remarks, we find the same writer, when speaking of two diseases on which medical men are most frequently consulted, acknowledge the impossibility of distinguishing either of them from almost any of the numberless appearances enumerated throughout the whole of his book. Speaking of the itch, he says,

"This troublesome disease, which, from its affinity with three orders of eruptive appearances—pustules, vesicles, and papulæ, almost bids defiance to any attempt to reduce it to an artificial classification, is not easily characterized in few words."

And afterwards—

"In the course (says he,) of this synopsis, I have made only cursory allusions to a very important class of cutaneous eruptions, which are often the source of considerable embarrassment to the practitioner; I mean those which are the result of the venereal poison. The subject, indeed, is difficult, and not yet sufficiently investigated; for these eruptions assume such a variety of forms, that they bid defiance to arrangement according to their external character; and, in fact, they possess no common or exclusive marks, by which their nature and origin are indicated."

Yet, it is certain, that these eruptions have been reduced to laws since the days of Mr. Hunter. We are ready to admit, that to comprehend the whole, requires that minute and candid attention which can only be expected in enlightened ened and honest minds. But we find they were sufficient to enable Mr. Wilson, surgeon of the Porpoise,* satisfactorily to determine, that syphilis did not exist in Otaheite. In this decision too, he was anticipated by a physician who could only collect the imperfect descriptions of other travellers. On all these accounts we applaud the prudence of Dr. Bateman in omitting, as we are informed he has done, in his edition just published, all that is contained in the former on the subject of syphilis. But still the same difficulties and uncertainties must continue, unless some discrimination is introduced in every other part of the new edition, because, in the former, the author tells us that "there is, perhaps, no order of cutaneous appearances, and scarcely any genus or species of the chronic eruptions, already described, which these secondary symptoms of syphilis do not occasionally resemble."

So much for the "Orders of cutaneous appearances, and the genera and species of chronic eruptions." We shall now add a few words on the merits of artists. In France they seem greatly to exceed ours, or "M. Alibert has indeed been particularly fortunate in those he employed." We are led to these reflexions by a third case of Elephantiasis† which has occurred in England, and an account of which is given in the last number but one of the Medico-Chirurgical Journal. The description is exceedingly accurate, but the engraving depicts a countenance more like a freckled sun-burnt clown of our northern climate, than of one labouring under lepra arabum. We are ready to admit that the disease, though chronic, varies in its external appearance, but less than almost any other; and, if even this cannot be faithfully represented in a single engraving, how can we expect it in any other still more variable disease? Happily, however, there are diagnostic symptoms unconnected with visage, which we transcribe from Dr. Lee's account.

---

* See our Journal, vol. xvi. p. 172; and Edin. Journ. vol. ii. p. 274, and vol. viii. p. 157, and p. 146.

† See our Journal, vol. xxxiii. p. 107 *et alibi.*

"The

"The features are very much swollen and deformed, and on the cheeks, nose, lips, and chin, as low down as the os hyoides, there are hard tuberculated elevations of the cutis. These tubercles on the cheeks and chin are so crowded together, as to form large, irregular, and, in some places, rather deep-seated lumps; and over these are placed a few smaller prominent and rounded tubercles, about the size of a garden pea; the whole presenting an appearance very similar to the rough tuberous elevations occasionally observed in potato-peel. These masses have an unctuous appearance and feeling, are of a dark brown or dusky colour; but those apparently more superficial are of a lighter and shining complexion, with some small florid blood-vessels ramifying on their surface. The temples retain their natural aspect, but on the forehead, which is very much wrinkled, there are situated a considerable number of flattened tubercles, varying in size from one to two lines, with general thickenings of the skin. The superciliary ridges are more than usually prominent, and the hairs are few and scattered. The upper eye-lids are thickened and tuberculated, but the cilia remain, and are of their usual appearance. The ears, particularly the lobes, helix, and anti-helix, are enlarged, and occupied by a number of small tubercles."*

We transcribe the following extract from a paper which appeared in our Journal, vol. xxiii. p. 107.

"In the upper and anterior part of each thigh there is a cluster of enlarged lymphatic glands, forming a moveable, rather soft, prominent swelling; in the left, about the size of a small hen's egg, without any discolouration of the integuments, or any pain."*

"The organs of generation appear to be nearly in the same condition as they are usually observed to be in boys before the age of puberty, with the exception of the scrotum, which, though nearly empty, is considerably larger. The testicles are small and soft, and are situated high up, near the external abdominal aperture. He reports that they have diminished nearly one half in size, and are still becoming smaller. There are no hairs on the pubis or chin. The prepuce is covered, both on the inner and outer surface, with tubercles, and is so much contracted that the glans cannot be completely uncovered.

"The disease, in this case, does not appear to be infectious, as

* Medico-Chirurgical Journal, p. 361.

this this boy has slept in the same bed with his brother during the whole period that he has been affected with the complaint, and in him none of the symptoms have occurred."

We shall now conclude the subject of engravings by transcribing a passage from the introduction to the last edition of Mr. Stevenson's Treatise on Cataract.

"The same disease, (says this gentleman,) in different persons, and in the same person at different times, varies so much as to baffle every effort to

"Catch 'ere she change the Cynthia of this minute."

Mr. Stevenson proceeds to remark, that, to convey to the eye a correct idea of such diseases, a number of plates would be necessary, equal to the changes the same disease undergoes.

That most pitiable class of patients, many of whom have hitherto been considered almost out of the pale of medicine, have lately met with the attention to which their unfortunate condition entitles them. Not only have physicians of every description been diligent in inquiring into the causes and mode of relieving what were considered maladies of the mind, but the legislature has taken the objects under their more immediate protection. Hitherto, however, not much light has been cast upon the medical arrangement of deranged intellect, although much ingenuity has been called into exercise upon this most interesting subject. It remains to be proved how far the new organology of Dr. Spurzheim will be brought to bear practically on this point. In the mean time we have to announce, from the pen of this author, an interesting work on "the Diseased Manifestations of the Mind." Dr. S. first treats of the disorders of the "*external* functions of the mind," including convulsions, epilepsy, catalepsy, and palsy; and then proceeds to notice the *internal* derangements,—under which division he includes apoplexy, phrenitis, hydrocephalus, and, lastly, insanity—its causes, varieties, prognosis, and treatment. In this last division of the subject, Dr. S., with propriety, objects to the empirical empirical practice of treating mental affections as always of a similar nature, and urges the necessity of attending, as well to the part of the brain affected, as to the kind of affection present, and to the constitution and former habits of the sufferer.

Haslam will always be a name of authority in relation to mental disorders. His last pamphlet will be perused with interest by such as are in possession of his former publications: there is nothing, however, of much novelty in the tract to which we now allude, on "the Moral Management of Insane Persons," which, indeed, is a mere expansion of a section in one of his former publications.

Two pamphlets have appeared on the subject of Mr. Rose's Bill, as it is called, the one by an anonymous author, entitled "Observations on the Laws relating to Private Lunatic Asylums," &c.; the other by Dr. Burrows, which takes nearly the same ground, and advances the same objections to the legislative act, framed in consequence of the late investigations, by a Committee of the House of Commons, into the abuses of private and public receptacles for the insane. The scope and tenor of these pamphlets are to show that the severity of the projected laws, while they will bear hard upon the managers of institutions for the insane, will necessarily prove destructive of their own intent. Our legislators, we think, ought to pay due attention to every thing in the way of stricture which comes from such respectable sources as those now alluded to. Both the pamphlets are productions of able writers and practical men.

"On Hypochondriasis and Nervous Affections," Dr. Reid has published a small volume, which cannot fail to command and rivet the attention of its readers by the force and fascination of its style. It is, however, rather a popular than a strict medical production, and does not profess to go into the minutiæ of pathological doctrines.

Mr. Parkinson has published a small pamphlet on the "Shaking Palsy," which he supposes to have an origin in the cervical part of the spinal medulla. He very modestly apologises for the hypothetical nature of his speculations on this this head. In this we think he hardly does himself justice. At the same time, he appears to us to have marked only one cause for a variety of diseases arising from various causes; and exhibiting different phenomena. The importance of his remarks, however, demand an early attention in our analytical department.

Dr. Crawford's posthumous publication on Tonic Medicines cannot fail to claim some share of interest, were it only as the last public legacy of this lamented benefactor to science. What we conceive to be its merits and defects have been so largely dwelt upon in the analytical department of our Journal, as to preclude the necessity of any further notice.

Dr. Balfour has complained of our review of his work on Percussion, &c. in Rheumatism. Our readers have seen his appeal against this supposed injustice on the part of our reviewer; and he will allow that we are not deficient in candour, when we admit that we have, since this controversy, witnessed, upon a small scale, the beneficial effects of the plan recommended in rheumatic affections by this author. [It may be some satisfaction to Dr. Balfour to be informed, that this *amende honorable* is from the writer in our Journal, by whom the doctor thought himself too severely treated.]

If the medical literature of Britain be, in any respect, inferior to that of our continental neighbours, it is, we think, in the article of medical jurisprudence. A good work on this subject, we deem to be still a desideratum. In the mean time, we have to notice a small compilation by Dr. Male, entitled, "An Epitome of Juridical or Forensic Medicine," which principally treats of the morbid symptoms produced by different poisons, and the means by which such poisons may be detected. The subject too of infanticide, suspended animation by drowning or hanging, insanity, pretended and imputed diseases, rape, impotence, &c. are slightly touched on. The author does not profess to have given more than a general outline of these particulars, which he has confessedly extracted from those works of most authority in which they are severally treated. This book has the same fault as we discovered in Dr. Farre's, namely, an inattention to the

 digestion digestion of the stomach after death. Whenever a well-digested work of medical jurisprudence is accomplished, we conceive it must be the joint labour of the two professions. A lawyer must point out the cases and explain the difficulties which have occurred to the court in each.

At the College of Physicians, and at the College of Surgeons, lectures are, at this moment, in the course of delivery. That the lecturer, in one instance, should read to the walls and benches, and, in the other, to crowded audiences, is among the many proofs how much the more showy branches of science are preferred. As Mr. Abernethy's introductory lectures will probably be published, we shall not anticipate any part of them. It is but bare justice to Dr. Yeats and Dr. Powell, the Croonian and Gulstonian lecturers of the present year in the College of Physicians, to state, that the lectures which they read merited a very different fate from what they received. The one series (Dr. Y.'s) was on the affections of the duodenum; and the other, (Dr. P.'s) on hæmorrhagic disorders of the encephalon and its enveloping membranes.

Dr. Yeats, after delineating in a very correct and interesting manner the separate and connected anatomy of the duodenum, commented upon the physiological office of this intestine, described the second digestion which the aliment undergoes in this part, and the important sympathies between it and the nervous system. He then entered upon the diseased conditions and actions of the duodenum, showing, that several symptoms which are often vaguely and indiscriminately referred to the stomach and liver, are, in reality, indications of duodenal disease, and are remedied with more facility and safety by alterative purgatives combined with vegetable tonics, than by the mercurial plan in common use. The dissertation was comprised in three lectures,—the number, we suppose, directed by their founder.

Dr. Powell immediately succeeded to Dr. Yeats, and began by stating his intention, as far as the limits of the lectures would permit him, to discourse on some of the most important points in the pathology of the brain. He very

 properly properly objected to the generic term of apoplexy, as including under one sweeping denomination, so many complaints of a widely diverse origin and nature. He then proceeded to describe the anatomy of the brain, principally in reference to its blood vessels; entered upon the theory of hæmorrhagic discharges ; and detailed, from his own practice, and from the works of the most accredited authors, instances of hæmorrhages in the brain illustrative of his pathological notions. To Dr. Powell succeeded Dr. Curry, who read a short lecture on the physiology of muscular action; but, as these notices must go to press before the conclusion of this last division of the series, we forbear at present any further observations on Dr. Curry's share in the series of discourses, which, we repeat, were pregnant with too much interest to have been treated on the part of the fellows and licentiates of the college with such marked neglect. If any observations of ours could tend to excite a disposition in future to attendance, we should feel a consciousness of having performed a public service.

On the whole, we feel much cause for satisfaction in having been induced to undertake this Retrospect. The hint came, it is true, from a suspicious quarter, and was probably thrown out more at random than with any knowledge. It has, however, given us an opportunity of wandering somewhat beyond our own limits. Not contented with alternate compliments and gentle chidings, couched in language so smooth and so light as to pass off without leaving an impression, and tending rather to relieve the reader from thinking than to call forth his intellectual powers, we have undertaken to point out those ancient land-marks beyond which medicine has advanced, and seems to promise a future progress,—sometimes with advantage, sometimes with doubtful issue, sometimes into labyrinths from which, with all the pains we are perpetually taking, we find it difficult to recover our own footsteps. It will probably be discovered, that the most important result of all these improvements is the confirmation it affords that medicine, like

 all all other sciences, is founded in good sense, and not in hypothesis. That it has its own laws, which are to be discovered only by watching all the operations of Nature in a living body, in health or under disease, and those changes which take place on the application or abstraction of various stimuli—dietetic, pharmaceutic, or mechanic. If the difficulties seem greater than in other arts, it is only because we anticipate conclusions, instead of accumulating and comparing facts; and because we forget that every fact is an experiment infinitely more conclusive than any we can artificially make. Often have we to lament that events the most common, and, on that account, the most important, either pass unnoticed, or are described with all the mysterious air of a discovery or of a theory. At other times, we find the application of artificial chemistry to produce what can only be accomplished by a secretion during life; for it cannot escape the most superficial reasoner, that, if the laws of chemical combination make any part of the animal economy, they must be directed with a nicety which no human power can imitate. In a word, every discovery we make tends to prove that there are no laws common to living and dead matter, excepting elasticity; and that even this, as long as life continues, is always controlled by the living powers.

This conviction has gradually produced a change in our medical language, first introduced by Mr. Hunter, and which requires all the foresight and sagacity of that great physiologist to keep it in order. We learn by degrees the importance of words, and, in proportion as we do so, we fall into a similar language. But let us never forget that words must be considered only as the means of communicating our thoughts with logical precision; and that it is previously necessary to digest those thoughts by a correct attention to facts, their series and order. For want of this, we have just seen one writer who, on a former occasion, treated some of Mr. Hunter's discoveries with the flippancy of ridicule, now anxious to adopt his language without taking the trouble of studying his doctrines, like a recruit who ventures to fire his piece before he is complete in the drill. This is particularly inconvenient in a didactic writer, because he never can explain his own expressions; nor is there any authority to which his reader can refer. In the writings of Dr. Parry, probably once a disciple of Mr. Hunter, we see a nearer approach to his mode of reasoning. But, even with him, a facility of writing has sometimes betrayed the scholar into expressions which might and ought to have been more guarded. Probably he might wish to avoid that incumbrance which, in Mr. Hunter's great caution, sometimes renders his reasoning obscure, and always much diminishes the interest felt by the reader. As we consider language not only the means but the test of improved reasoning, we might illustrate our meaning by an extract from each of these writers. But, having so lately gone over Dr. Parry's ingenious work, we shall only trouble the reader with a specimen of Mr. Hunter.

"I shall carry my ideas of life (says he,) further than has commonly been done: life I believe to exist in every part of an animal body, and to render it susceptible of impressions which excite action; there is no part which has not more or less of this principle, and consequently no part which does not act according to the nature of the principle itself, and the impressions thence arising, producing thereby infinite variety, both in all natural and diseased acts. How far every part has an equal quantity of life, or of the powers of life, is not easily ascertained; but, if we were to estimate them by the powers of action, we should judge tolerably well. Disease would seem to give some intelligence with regard to this matter; but how far resistance to disease, and powers of restoration, depend on the powers of life, or simply on the powers of action, I cannot say; but I believe it may be set down as a rule, that those parts that are endowed with most action resist disease most strongly, and in disease restore themselves more readily to a healthy state."

This passage (from the introduction to Mr. Hunter's grand work), however awkwardly expressed, affords a just illustration of the manner in which all his reasoning is confined to life, its powers, and its actions, from different stimuli and sympathies, under disease and in health.

And

And now to return to, as we began with, our friend Juvenis. Let not his gentle ear, accustomed to the blandishment of a smoother style, revolt at the above. Nor should he be alarmed if he cannot immediately feel an interest, or perhaps comprehend a dull introduction to a series of abstract reasoning. As well might the school-boy, immediately on matriculation at the university, expect to read Locke's Essay on the Human Understanding with interest, or readily to comprehend it without a tutor. At first, we would advise Juvenis to confine his reading to collections of cases. These, from the interest they excite, will remain with him hereafter, when he may have occasion to apply them to pathological reasoning. If his master's library furnishes the Edinburgh Medical Essays, he cannot begin with a better compilation; they were principally collected by that enlightened and conscienscious professor, the first Dr. Alexander Monro, and contain many of his original papers. The abridgment in two volumes, for a learner, is the most desirable. In some of the professor's papers will be found the first dawnings of that improved pathology which it was reserved for his illustrious countryman, the late John Hunter, to advance nearer to a full meridian. A happy talent at investigation taught each to study nature only; so that, if a student should not immediately comprehend their reasoning, he will not be led astray by pursuing a devious path. And at all ages it should be remembered—

"Errores radicales in prima digestione mentis, &c."

Every other miscellaneous collection, particularly those of the College of Physicians, and various societies, will be perused with great advantage.

Another invaluable source of profitable amusement, found in almost every medical library, is Vanswieten's Commentaries. In these, the student will meet with all the knowledge and all the gossip previous to the æra in which this honest and industrious compiler wrote. We would, however, rather recommend the later volumes. Juvenis may open any one of them, and read on as he would a novel, with with as much entertainment and more advantage. If the library furnishes a Latin copy, he may at the same time acquire a greater facility at medical Latin, which, though not always elegant, is never barbarous. Whilst we recommend the above works to our younger readers, it cannot be supposed that we mean to consider them unworthy the notice of our equals in age. In truth, we conceive that few people fail to consult such an oracle as Vanswieten, whenever they wish a reference to every preceding writer on almost any subject in the various branches of medicine. Such, we are ready to acknowledge is often our own custom, and it is seldom that we close the volume unsatisfied in these particulars. It is a serious evil in modern medical education, that men read too little. This arises partly from their education commencing at a time, and under circumstances, which allow no leisure for reading. Apprentices, not incumbered with the interruption of an open shop, have advantages of which they rarely avail themselves, and they cannot too often be reminded that such opportunities may never occur in after-life.

