# Errata

**Published:** 1817-07

**Authors:** 


ERRATA.

In page 1, the learned reader will detect the want of prosody, and recollect the word that should be substituted for *ament.*

Page 10, for *septiunt* read *sentiunt.*

